# Translation and Validation of the Bangla Version of the Osteoporosis Knowledge Assessment Tool (OKAT)

**DOI:** 10.7759/cureus.105080

**Published:** 2026-03-11

**Authors:** Md. Rezaul Karim, Md. Nazrul Islam, A. K. Ahmedullah, Asif Hasan Khan, Sudeshna Sinha Trina, M Sheikh Giash Uddin, Jesmine Ara, Tamanna Tabassum, Jannatul Fardous

**Affiliations:** 1 Department of Rheumatology, Sylhet MAG Osmani Medical College, Sylhet, BGD; 2 Rheumatology, Bangladesh Medical University, Dhaka, BGD; 3 Rheumatology, Rajshahi Medical College, Rajshahi, BGD; 4 Department of Statistics, Jagannath University, Dhaka, BGD; 5 Department of Bangla, Mirpur Girls Ideal College, Dhaka, BGD; 6 Public Health, Pi Research and Development Center, Dhaka, BGD; 7 Psychiatry, National Institute of Mental Health, Dhaka, BGD

**Keywords:** bangla, bangladesh, osteoporosis, osteoporosis knowledge assessment tool (okat), psychometric validation

## Abstract

Objective: This study aimed to translate and validate the Osteoporosis Knowledge Assessment Tool (OKAT) for Bangla-speaking populations in Bangladesh by assessing its internal consistency, test-retest reliability, content validity, and construct validity.

Methods: This methodological validation study was conducted over 22 months in the Department of Rheumatology at Bangladesh Medical University, Dhaka. The study comprised two phases. The first phase included the translation of the OKAT questionnaire into Bangla (OKAT-Bangla), and the second phase included the psychometric validation of the OKAT-Bangla. A total of 126 individuals attending the study department were purposively selected as study participants. Data collection was conducted via a case record form. Each participant was subjected to a face‒to‒face interview followed by a retest after 1 week. To validate the OKAT-Bangla, internal consistency and reliability were tested along with content and construct validation. Statistical analysis was conducted via IBM SPSS Statistics for Windows, version 23.0.

Results: In this study, OKAT-Bangla demonstrated internal consistency with Cronbach’s alpha (α) of 0.636 and test‒retest reliability with a correlation coefficient (r) ranging from 0.55 to 0.94. Content validity was tested by expert rheumatologists, with an item-level content validation index of 1 and a scale-level content validation index of 1, indicating excellent relevancy. Principal component analysis revealed nine components with eigenvalues >1. The rotation sums of the squared loading variances ranged from 6.184% to 8.060%. These findings collectively establish the OKAT-Bangla as a valid instrument for measuring osteoporosis knowledge.

Conclusion: The OKAT-Bangla is a valid, reliable, and culturally appropriate instrument for assessing osteoporosis knowledge in Bangla-speaking populations.

## Introduction

Osteoporosis is a systemic disorder that primarily affects the skeletal system and is characterized by worsening bone mineral density with an increased risk of fracture [1]. It is a progressive condition that significantly impacts quality of life and is associated with morbidity and mortality worldwide [2]. Although osteoporosis cannot be completely cured, its progression can be slowed through preventive measures such as lifestyle modifications and dietary adjustments [3,4]. Health education plays a crucial role in preventing osteoporosis, making it essential to understand the existing knowledge about the disease in the community to develop effective educational interventions [5,6].

Preventive measures for osteoporosis include increasing physical activity, quitting smoking, limiting alcohol consumption, and ensuring adequate intake of calcium and vitamin D [7-9]. To maximize the adoption of these measures, community-based health promotion strategies must be designed and implemented on the basis of an accurate assessment of the community’s overall knowledge level about osteoporosis. Assessment of knowledge level requires standardized and reliable tools to ensure accurate and meaningful evaluation. Various tools have been developed to measure osteoporosis knowledge, including the Facts on Osteoporosis Quiz (FOOQ), the Osteoporosis Questionnaire (OPQ), the Osteoporosis Knowledge Test (OKT), and the Osteoporosis Knowledge Assessment Tool (OKAT) [10-13]. Among these, the OKAT is recognized for its comprehensive approach to evaluating individual understanding of osteoporosis, associated risk factors, symptoms, preventive measures, and management options [14].

The OKAT was originally developed in English to assess osteoporosis knowledge among Australian women [13]. Since its inception, it has been utilized globally and validated in several languages, demonstrating its versatility and applicability across different cultural contexts [15-20]. However, until now, there has been no validated Bangla version of the OKAT for Bangla-speaking communities in Bangladesh. Validating the OKAT in the native language is crucial to ensure accurate application and interpretation of this tool. Bangladesh has a large Bangla-speaking population, and without a validated tool in their native language, assessing the overall understanding of this population is challenging.

The prevalence of osteoporosis in Bangladesh is 35-37%, which is notably high, particularly among postmenopausal women [21,22]. Despite the significant burden of the disease, there is a lack of data on the general population’s knowledge about osteoporosis in Bangladesh. To develop effective national awareness and prevention programs, it is imperative to assess the current level of knowledge within these communities. Therefore, this study aimed to translate, culturally adapt, and validate the original OKAT tool for Bangla-speaking populations in Bangladesh. The specific objectives were to translate a Bangla version of the OKAT using a standardized forward-backward translation process, then evaluate its internal consistency and test-retest reliability, establish content, and construct validity.

A Bangla version of the OKAT might ensure the development of educational and preventive measures that can be effectively tailored to meet the specific needs of Bangla-speaking communities. Moreover, a culturally adapted and validated tool might enhance the reliability of the data collected, leading to more effective and targeted interventions. By providing a localized version of the OKAT, this study not only facilitates the assessment of osteoporosis knowledge but also lays the foundation for targeted educational programs aimed at improving bone health and reducing the incidence of osteoporosis-related fractures in Bangladesh.

## Materials and methods

Study design, location, and period

This methodological validation study was conducted in the Department of Rheumatology at Bangladesh Medical University (BMU), Dhaka, over a period of 22 months from May 2016 to March 2018.

The study was conducted through two phases. Phase 1 involved the translation of the original English OKAT questionnaire to Bangla, followed by pretesting. Phase 2 involved the psychometric validation of the Bangla version of the OKAT (OKAT-Bangla).

Structure of the OKAT questionnaire

The original OKAT questionnaire (Appendix I) contains 20 questions: the first 12 assess knowledge about osteoporosis, the next four evaluate attitudes toward the condition, and the final four assess practices and perceptions regarding prevention. Responses are scored as "true," "false," or "unknown," with "true" earning one point and the others earning zero points. The total summed scores are categorized as low (0-7), moderate (8-14), or high (15-20) knowledge of osteoporosis.

Phase 1

Translation: In Phase 1, the original English OKAT was translated and backtranslated following the steps suggested by Beaton et al. [23]. Two native Bangla speakers conducted separate forward translations, which were synthesized by experts and the research team and then back-translated to English by two other translators unaware of the original version. An expert committee, including rheumatologists and language professionals, reviewed all the versions and constructed a pre-final version of OKAT-Bangla to ensure semantic, idiomatic, experiential, and conceptual equivalence.

Pretesting, understandability, and cognitive briefing: To ensure understandability, the pre-final version of the OKAT-Bangla was tested with 20 school children (10 boys and 10 girls) aged 12 years. Each question was assessed for clarity, and any difficult terms were revised on the basis of their feedback. Then, the pre-final version was pretested involving 40 adults aged 18 years and above from the rheumatology department at the BMU. The participants were interviewed to gauge their comprehension of each item and their responses. The feedback from these sessions was used to refine the questionnaire further, ensuring that it retained its equivalence to the original in applied situations, and thus the OKAT-Bangla was finalized (Appendix II).

In this phase, sample sizes are picked following established guidelines: five to 15 (or up to 20 for pediatric groups) for cognitive debriefing to reach saturation in problem detection, and 30-50 for adult pretesting to assess applied comprehension with adequate feasibility [24,25].

Phase 2

Study population, sample size, and data collection procedure: The study population comprised patients and healthy attendants visiting the Department of Rheumatology, BMU. The sample size was determined based on an item-to-sample ratio of 1:7. Since the OKAT tool consists of 20 items, the calculated sample size was 140. Participants were purposively selected from consecutive patients and attendants visiting the department who met the inclusion criteria.

So, a total of 140 adult individuals, aged ≥18 years, were recruited for psychometric validation. Inclusion criteria included the ability to read and write in Bangla, willingness to provide written informed consent, and no cognitive impairment affecting questionnaire completion. The participants were informed about the study objectives and provided written consent. The exclusion criteria included severe illness, mental retardation, handicapped individuals, illiteracy, and inability to self-administer questionnaires.

Data collection was facilitated via a self-administered structured case record form that included sociodemographic information and two sets of the OKAT-Bangla questionnaire, each marked with an identifying number. All participants were subjected to a test and then retested after seven days. Participants who could not attend the retest were excluded from the final analysis.

Participants with incomplete responses on either questionnaire administration (n = 14) were excluded from psychometric analyses to ensure data quality and avoid bias from partial responses. No imputation methods were applied due to the small number of missing cases. A complete case analysis was conducted. Finally, a total of 126 study participants were analyzed.

All participants completed two administrations of the OKAT-Bangla questionnaire with a seven-day test-retest interval. The seven-day interval was selected to minimize recall bias while ensuring stability of the construct (osteoporosis knowledge). Questionnaires were self-administered using a structured case record form with identical item order maintained across both administrations. Participants responded independently without external assistance or discussion. Data were collected by trained research staff following standardized protocols. Collected data were manually entered into Microsoft Excel (Microsoft Corp., USA) and the IBM SPSS Statistics database to ensure data integrity. This approach enhances reproducibility and minimizes measurement error.

Psychometric validation: For psychometric validation, the internal consistency and test‒retest reliability of the OKAT-Bangla were assessed from 126 complete responses. The participants completed the questionnaire twice, with a seven-day interval for the retest. Internal consistency was measured via Cronbach’s alpha, and reliability was assessed via test‒retest correlation. Content validity was evaluated via item-level (I-CVI) and scale-level (S-CVI) content validity indices, which were rated by four rheumatologists. The construct validity was demonstrated via factor analysis (principal component analysis).

Statistical analysis

Statistical analysis was performed via IBM SPSS Statistics for Windows, Version 21.0 (released 2015, IBM Corp., Armonk, NY). A p-value of <0.05 was considered to indicate statistical significance. Content validity was assessed by the item-level content validity index (I-CVI) and the scale-level content validity index. Internal consistency was measured by Cronbach’s alpha (α). Test‒retest reliability was assessed by demonstrating the correlation between the test responses and retest responses via the intraclass correlation coefficient (r). Factor analysis was conducted through principal component analysis with Varimax rotation to enhance interpretability. The Kaiser-Meyer-Olkin (KMO) measure of sampling adequacy and Bartlett’s test of sphericity were assessed to verify the appropriateness of factor analysis for this dataset. Components with eigenvalues exceeding 1.0 were extracted. Factor loadings ≥0.4 were considered meaningful; loadings <0.4 were interpreted as weak associations.

Ethical considerations

All procedures in this study involving human participants were performed in accordance with the ethical standards of the institutional and/or national research committee and in accordance with the Declaration of Helsinki. The study was approved by the Institutional Review Board of Bangladesh Medical University (Formal Bangabandhu Sheikh Mujib Medical University) (BSMMU/2017/1237). Permission to translate and use the OKAT questionnaire was obtained from the originator.

## Results

The majority of the study participants were 18-27 years of age, with a mean age of approximately 31.60 ± 10.22 (SD) years. There was an equal distribution of males and females, with the vast majority (95.2%) residing in urban areas. More than half of the study patients completed graduation or postgraduation as their highest educational attainment. With respect to occupation, the majority were students, followed by housewives and private service employees (Table 1).

**Table 1:** Sociodemographic characteristics of the study participants (n = 126) Note: The mean age was 31.60 ± 10.22 years.

Variable	n	%
Age group (years)		
18-27	50	39.7
28-37	44	34.9
38-47	19	15.1
≥48	13	10.3
Gender		
Male	63	50.0
Female	63	50.0
Residence		
Rural	6	4.8
Urban	120	95.2
Highest educational attainment		
Secondary school	11	8.7
Higher secondary college	47	37.3
Graduation/higher	68	54.0
Occupation		
Housewife	32	25.4
Government service holder	10	7.9
Private service holder	30	23.8
Businessman	7	5.6
Farmer	1	0.8
Student	43	34.1
Others	3	2.4

The mean total OKAT score was 8.53 ± 3.0 (SD). The corrected item‒total correlations ranged from -0.04 (Q14) to 0.44 (Q11), indicating weak to moderate correlations between individual components and the overall OKAT score. The test‒retest reliability coefficients ranged from 0.55 (Q12) to 0.94 (Q20), indicating moderate to strong consistency of the responses over time (Table 2).

**Table 2:** Descriptive and reliability statistics of the Osteoporosis Knowledge Assessment Tool (OKAT) Bangla Test‒retest reliability was determined via the Pearson correlation test.

Question no.	Mean±SD	Corrected item-total correlation	Test-retest reliability ®	Cronbach’s alpha (α) if the item was deleted
Q1	0.93 ± 0.26	0.32	0.66	0.62
Q2	0.22 ± 0.42	0.30	0.76	0.62
Q3	0.20 ± 0.40	0.37	0.58	0.61
Q4	0.51 ± 0.50	0.26	0.91	0.62
Q5	0.64 ± 0.48	0.30	0.78	0.61
Q6	0.33 ± 0.47	0.36	0.86	0.61
Q7	0.63 ± 0.49	0.22	0.81	0.63
Q8	0.75 ± 0.44	0.32	0.83	0.61
Q9	0.32 ± 0.47	0.41	0.67	0.60
Q10	0.35 ± 0.48	0.17	0.72	0.63
Q11	0.10 ± 0.30	0.44	0.65	0.61
Q12	0.37 ± 0.49	0.36	0.55	0.61
Q13	0.11 ± 0.32	0.35	0.66	0.61
Q14	0.79 ± 0.41	-0.04	0.67	0.65
Q15	0.15 ± 0.36	0.04	0.61	0.64
Q16	0.63 ± 0.49	0.08	0.81	0.64
Q17	0.55 ± 0.50	0.18	0.84	0.63
Q18	0.06 ± 0.23	0.07	0.66	0.64
Q19	0.19 ± 0.39	0.14	0.84	0.63
Q20	0.74 ± 0.44	-0.02	0.94	0.65
Total	8.53 ± 3.0			0.636 (overall)

The overall internal consistency of the OKAT-Bangla tool was moderate, with a Cronbach’s alpha of 0.636 (Table 2).

A content validity test involving four expert rheumatologists revealed item-level and scale-level content validity indices of 1.0 across all the items (Table 3).

**Table 3:** Content validity of the Osteoporosis Knowledge Assessment Tool (OKAT) Bangla

Content validity test	Findings
Item-level content validity index (I-CVI)	1
Scale-level content validity index (S-CVI)	1

Factor analysis suitability was confirmed by the Kaiser-Meyer-Olkin (KMO) measure (>0.6) and significant Bartlett's test of sphericity (p < 0.001), as detailed in the Methods section. Principal component analysis identified nine components with eigenvalues >1 (ranging from 1.004 to 2.214). The relatively equal distribution of variance across components (6.184-8.060%) demonstrated that each component captured meaningful variability, with no single dominant factor. Each component demonstrated clear theoretical interpretability, corresponding to distinct osteoporosis knowledge domains: (1) disease pathophysiology, (2) epidemiology and prevalence, (3) risk factors, (4) clinical presentation, (5) diagnostic methods, (6) prevention strategies, (7) treatment modalities, (8) behavioral practices, and (9) attitudes and perceptions (Table 4).

**Table 4:** Principal component analysis showing initial eigenvalues and rotation sums of squared loadings for the OKAT-Bangla Extraction method: principal component analysis

Component	Initial eigenvalues total	Rotation sums of squared loadings % of variance
1	2.214	8.060
2	1.822	7.926
3	1.569	7.427
4	1.424	7.214
5	1.347	7.109
6	1.282	6.912
7	1.116	6.860
8	1.050	6.447
9	1.004	6.184

Factor analysis was conducted. The questions demonstrating the strongest associations with their components included Q5 (osteoporosis leads to an increased risk of bone fractures), with a loading of 0.708 (Component 1), and Q17 (cigarette smoking can contribute to osteoporosis), with a loading of 0.667 (Component 1), representing fundamental osteoporosis concepts. Strong associations were also observed for questions such as Q4 (osteoporosis affects bone strength), with a loading of 0.694 (Component 2); Q2 (osteoporosis can be diagnosed through a bone density test), with a loading of 0.652 (Component 2); Q3 (low body weight is a risk factor for osteoporosis), with a loading of 0.683 (Component 3); Q6 (osteoporosis can cause a decrease in height), with a loading of 0.791 (Component 5); and Q9 (osteoporosis can be treated with medication), with a loading of 0.804 (Component 6). These strong loadings indicate core osteoporosis knowledge areas regarding disease manifestations, diagnosis, and treatment. Moderate associations were observed for Q8 (family history is a risk factor for osteoporosis), with a loading of 0.700 (Component 8); Q14 (osteoporosis can be prevented with adequate calcium intake), with a loading of 0.669 (Component 4); Q12 (men can develop osteoporosis), with a loading of 0.602 (Component 6); Q7 (lack of physical activity increases the risk of osteoporosis), with a loading of 0.539 (Component 9); Q11 (calcium supplements are recommended for people with osteoporosis), with a loading of 0.443 (Component 4); and Q1 (osteoporosis is common in women after menopause), with a loading of 0.777 (Component 9). These moderate loadings represent important applied and epidemiological knowledge. Conversely, the questions showing weaker associations included Q13 (vitamin D is important for bone health), with a loading of 0.663 (Component 7); Q15 (weight-bearing exercises help prevent osteoporosis), with a loading of 0.635 (Component 7); Q18 (hormone replacement therapy can be used to treat osteoporosis), with a loading of 0.694 (Component 3); Q16 (excessive alcohol consumption is a risk factor for osteoporosis), with a loading of 0.610 (Component 8); Q19 (osteoporosis can cause chronic pain), with a loading of 0.679 (Component 5); Q20 (there are no effective treatments for osteoporosis available in Bangladesh), with a loading of 0.437 (Component 8); and Q10 (any type of physical activity is beneficial for osteoporosis), with a loading of -0.566 (Component 1), indicating a negative association (Table 5).

**Table 5:** Structure matrix of the Osteoporosis Knowledge Assessment Tool (OKAT) Bangla showing the factor loading of each component

	Components								
Questions	1	2	3	4	5	6	7	8	9
Q1									0.777
Q2		0.652							
Q3			0.683						
Q4		0.694			-0.315				
Q5	0.708						0.354		
Q6					0.791				
Q7		-0.423							0.539
Q8								0.700	
Q9						0.804			
Q10	-0.566								
Q11		0.337	0.379	0.443			0.424		
Q12	0.315					0.602			
Q13							0.663		0.314
Q14				0.669					
Q15							0.635		
Q16		-0.472						0.610	
Q17	0.667			0.318					
Q18			0.694						
Q19					0.679	0.320			
Q20	-0.365	0.331	0.311					0.437	-0.437

## Discussion

This study conducted a standardized translation of the original OKAT into a Bangla version (OKAT-Bangla) and validated it for the Bangla-speaking community. The OKAT-Bangla demonstrated internal consistency with a Cronbach’s alpha of 0.64. which is slightly below the conventional threshold of 0.70 but comparable to the original English OKAT version (alpha = 0.69), demonstrating that we have accurately replicated the psychometric properties of the source instrument [13]. In contrast, the Hungarian and Indian versions exhibited excellent internal consistency, with Cronbach’s alpha values of 0.81 and 0.89, respectively [19,20]. The differences in cultural and contextual environments, such as varying levels of health awareness and education, may influence the consistency of responses in Bangladesh. In Bangladesh, public awareness of osteoporosis and general health literacy levels are often lower than those in more developed countries. This lack of awareness can lead to misunderstandings or misconceptions about the disease, its risk factors, and preventive measures.

The test-retest reliability of the OKAT-Bangla indicated moderate to strong consistency of responses over time, demonstrating that participants’ answers were stable across repeated administrations of the questionnaire. Overall, strong reliability was reported for the OKAT-Arabic version [26]. This consistency is crucial for establishing the reliability of a tool designed to measure knowledge over time.

The content validity of the OKAT-Bangla was rigorously evaluated by an expert panel of rheumatologists to ensure that the questionnaire items were relevant and appropriate for assessing osteoporosis knowledge in the Bangladeshi context. The item-level content validity index (I-CVI) and the scale-level content validity index (S-CVI) indicate high relevance of the entire set of items in the questionnaire and demonstrate that the OKAT-Bangla is a well-constructed tool with items that are pertinent and reflective of the key aspects of osteoporosis knowledge.

Principal component analysis revealed nine components, demonstrating the applicability of a multidimensional approach for the OKAT-Bangla. The structure matrix indicated variable factor loadings: questions with strong associations included Q5 (osteoporosis leads to an increased risk of bone fractures) and Q17 (cigarette smoking can contribute to osteoporosis), suggesting that these items are crucial for assessing key areas related to osteoporosis. Questions with moderate associations, such as Q8 (family history is a risk factor for osteoporosis), Q12 (men can develop osteoporosis), and Q14 (osteoporosis can be prevented with adequate calcium intake), highlight areas where knowledge is variable and requires further improvement. Conversely, questions with lower factor loadings indicate areas of poor understanding that need targeted educational efforts. Hence, the nine-component factor structure was justified through multiple complementary approaches beyond the Kaiser criterion (eigenvalue >1) alone. First, the relatively equal variance distribution across components indicated that each component represented meaningful and distinct variability, with no single component dominating the structure. Second, the theoretical interpretability of each of the nine components corresponding to distinct osteoporosis knowledge domains supported the multidimensional conceptualization of osteoporosis knowledge. Third, the pattern of factor loadings demonstrated coherent domain structure: This gradient pattern reflects the legitimate complexity of osteoporosis knowledge rather than representing statistical artifacts or measurement error.

The validated OKAT -Bangla is a vital tool for assessing osteoporosis knowledge among Bangla-speaking populations. It helps identify knowledge gaps and misconceptions, allowing healthcare providers to design targeted educational programs. By enhancing knowledge and awareness, these programs can promote preventive measures such as increased physical activity, adequate calcium and vitamin D intake, and avoidance of smoking and excessive alcohol consumption, ultimately helping to slow the progression of osteoporosis.

While lower alpha values might suggest weaker relationships among individual items, this does not necessarily indicate that the instrument fails to measure its intended construct. The internal consistency and test-retest reliability of OKAT-Bangla support measurement stability and indicate that the tool produces consistent responses over time. This finding is further supported by content validation and exploratory factor analysis. However, stronger evidence for construct validity would require convergent validation and criterion validation. These approaches would further strengthen evidence for construct validity beyond the factor structure demonstrated here. Also, the sample participants may not fully represent the diversity of the Bangladeshi population, as the study was conducted in a single department of a tertiary hospital in the capital city rather than involving participants from community-level settings. This hospital-based, urban-centric sampling approach may overrepresent individuals with higher health-seeking behavior and educational exposure, thereby limiting the generalizability of the findings to community-level and rural populations. Future studies should incorporate multicenter designs with community-based sampling across multiple geographic regions to improve external validity.

## Conclusions

The Bangla version of the OKAT demonstrated acceptable content validity, satisfactory internal structure, and good test-retest reliability in this study population. These findings support its preliminary use as a reliable instrument for assessing osteoporosis-related knowledge among Bengali-speaking individuals in clinical and research settings. Further studies are warranted to establish convergent validity, determine responsiveness to educational interventions, and evaluate its applicability across diverse healthcare settings and demographic groups. Broader validation will enhance generalizability and strengthen its utility for public health and preventive strategies in Bangladesh and other Bangla-speaking populations.

## Figures and Tables

**Figure 1:**
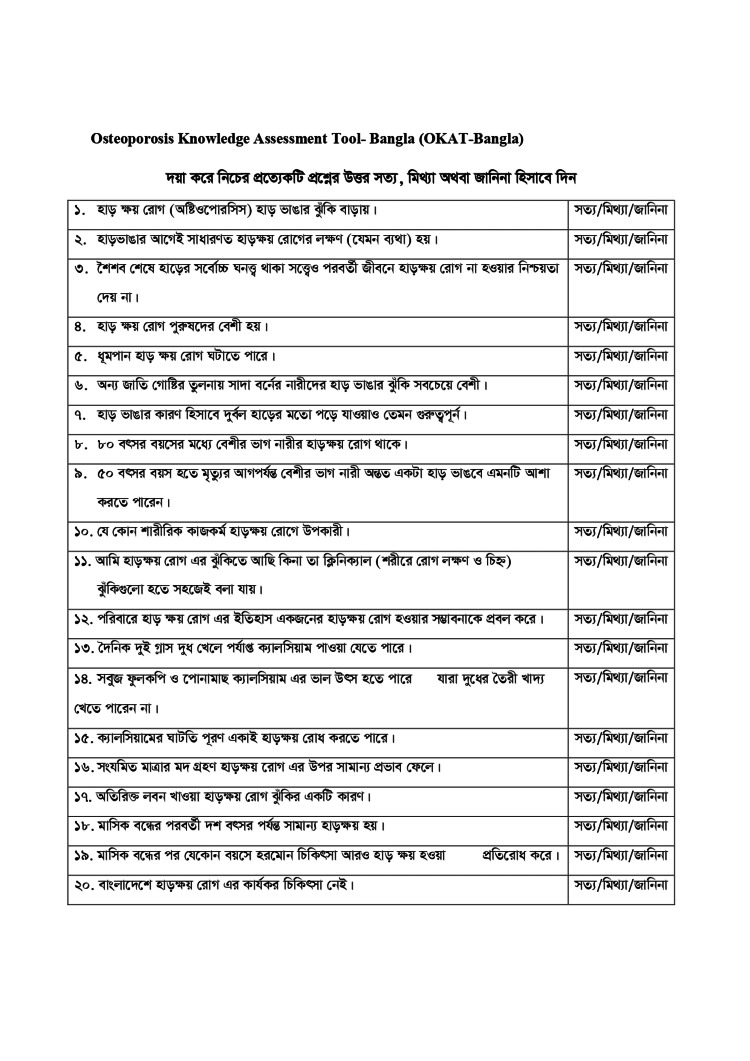
Osteoporosis Knowledge Assessment Tool-Bangla (OKAT-Bangla)

## Appendices

Appendix I 

**Table 6:** Osteoporosis Knowledge Assessment Tool (OKAT)

Please answer each of the following questions with True, False or Don’t Know.	
1. Osteoporosis leads to an increased risk of bone fractures.	True/false/don’t know
2. Osteoporosis usually causes symptoms (e.g. pain) before fractures occur	True/false/don’t know
3. Having a higher peak bone mass at the end of childhood gives no protection against the development of osteoporosis in later life.	True/false/don’t know
4. Osteoporosis is more common in men.	True/false/don’t know
5. Cigarette smoking can contribute to osteoporosis	True/false/don’t know
6. White women are at highest risk of fracture as compared to other races.	True/false/don’t know
7. A fall is just as important as low bone strength in causing fractures.	True/false/don’t know
8. By age 80, the majority of women have osteoporosis	True/false/don’t know
9. From age 50, most women can expect at least one fracture before they die	True/false/don’t know
10. Any type of physical activity is beneficial for osteoporosis	True/false/don’t know
11. It is easy to tell whether I am at risk of osteoporosis by my clinical risk factors	True/false/don’t know
12. Family history of osteoporosis strongly predisposes a person to osteoporosis	True/false/don’t know
13. An adequate calcium intake can be achieved from two glasses of milk a day.	True/false/don’t know
14. Sardines and broccoli are good sources of calcium for people who cannot take dairy products	True/false/don’t know
15. Calcium supplements alone can prevent bone loss.	True/false/don’t know
16. Alcohol in moderation has little effect on osteoporosis.	True/false/don’t know
17. A high salt intake is a risk factor for osteoporosis.	True/false/don’t know
18. There is a small amount of bone loss in the ten years following the onset of menopause.	True/false/don’t know
19. Hormone therapy prevents further bone loss at any age after menopause	True/false/don’t know
20. There are no effective treatments for osteoporosis available in Bangladesh	True/false/don’t know

Appendix II 
